# Effect of early life stress on anxiety and depressive behaviors in adolescent mice

**DOI:** 10.1002/brb3.1526

**Published:** 2020-01-21

**Authors:** Ting He, Chen Guo, Chunlian Wang, Chunrong Hu, Huanxin Chen

**Affiliations:** ^1^ Key Lab of Cognition and Personality of the Ministry of Education Collaborative Innovation Center for Brain Science School of Psychology Southwest University Chongqing China; ^2^ Department of Alternative Medicine No. 9 Chongqing Hospital Chongqing China

**Keywords:** adolescence, anxiety, depression, early life stress, sex difference

## Abstract

**Introduction:**

Adolescence is a critical period for physical and mental development. The effect of early life stress on mood disorders has been intensively studied in adults using rodent models, but it has been less studied in adolescents. The present study aimed to examine the effect of early life stress on anxiety‐related and depression‐like behaviors in adolescent C57BL/6 mice and the sex difference.

**Methods:**

C57BL/6 mice of both sexes were used, and early life stressors included maternal separation (MS, P2‐12, 4 hr per day), restraint stress (RS, P33 to 39, 4 hr per day), and their combination (MRS). Open field test, elevated plus maze, and forced swimming test were performed at different time points during adolescence and adulthood.

**Results:**

It was found that MS did not affect the anxiety‐related behaviors of both males and females tested on P30‐31 and P41‐42. RS decreased the anxiety level in adolescent males but did not affect it in the females. MS, RS, and MRS all significantly increased the depression‐like behavior in adolescent males, but only MRS increased the depression‐like behavior in adolescent females. All effects on adolescent males and females did not persist into adulthood.

**Conclusion:**

The present results showed that early life stress affected anxiety‐related and depression‐like behavior in adolescent mice in manners depending on the nature of stress, the developmental period, and sex.

## Graphical abstract

Early life stress affects anxiety and depressive behaviors in adolescent mice in a manner specific to sexes and stressors. These effects are transient and occurred during adolescence.

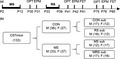

## 1 INTRODUCTION

Early life stress is strongly associated with mood disorders, such as depression and anxiety, in adolescence and adulthood (Chapman et al., 2004; Dougherty et al., 2013; Dunn, McLaughlin, Slopen, Rosand, & Smoller, 2013; Heim & Nemeroff, 2001; Hughes et al., 2017; Kessler et al., 2010). It also increases the risk of alcohol and substance use disorders (Dube, 2007; Dube et al., 2003). Adolescence is the critical period for physical and mental development and maturation, and especially vulnerable to chronic and adversary stress (Brenhouse & Andersen, 2011; Sturman & Moghaddam, 2011; Thapar, Collishaw, Pine, & Thapar, 2012; Watt, Weber, Davies, & Forster, 2017). The prevalence of depression and anxiety markedly increases after children grow to adolescence (Avenevoli, Swendsen, He, Burstein, & Merikangas, 2015; Bessdo, Knappe, & Pine, 2009; Dougherty et al., 2013; Mojtabai, Olfson, & Han, 2016). Moreover, abused adolescents show significantly higher rates of depression and behavior problems (Pelcovitz et al., 1994). Early life stress may alter neuronal networks of mood and cognitive systems, predisposing adolescents to developing psychological and psychiatric disorders that may continue to adulthood (Bonnie & Nim, 2015; Watt et al., 2017). However, the psychopathology of adolescents to early life stress is still not well understood.

Maternal separation (MS) in rodents, especially rats, has been widely used to elicit the effect of early life stress on mood disorders (Tractenberg et al., 2016; Vetulani, 2013). MS has been reported to increase anxiety and depressive behaviors and cause cognitive disorder in adulthood, which was accomplished by alterations in HPA axis, decreased BDNF expression and altered structural and functional plasticity in the medial prefrontal cortex and hippocampus in rats and mice (Hennessy, Schreibeis, Schiml, & Deak, 2017; Musazzi et al., 2009; Seo et al., 2017; Tractenberg et al., 2016; Vetulani, 2013; van Zyl, Dimatelis, & Russell, 2016). Though most mental disorders in humans often begin to onset during adolescence (Kessler et al., 2007), previous studies in rodent models, however, mostly focused on the adult outcomes of early life stress (Schroeder, Notaras, Du, & Hill, 2018). Only a few numbers of studies have directly examined the effect on adolescents, and the findings were varied and inconsistent. In adolescent C57BL/6 mice, some studies indicated that MS enhanced anxiety‐related and depression‐like behaviors (Bian et al., 2015), but others found it only increased anxiety‐related behaviors (Shin, Baek, Han, & Min, 2019; Shin, Han, Woo, Jang, & Min, 2016). In adolescent rats, MS has been shown to increase depressive behaviors during adolescence (Leussis, Freund, Brenhouse, Thompson, & Andersen, 2012; Lukkes, Meda, Thompson, Freund, & Andersen, 2017). However, reduced anxiety and depressive behaviors in adolescence after MS were also reported (Kwak et al., 2009; Wang, Li, Du, Shao, & Wang, 2015). The duration of MS appears to play a crucial role. It has been reported that short‐term MS (P1‐P10) did not affect anxiety but had an antidepressant effect in adolescents and adults. Long‐term MS (P1‐P21) increased anxiety and impaired recognition but did not change the depression‐like behavior in adolescence and adulthood (Banqueri, Méndez, & Arias, 2017). The sex is also an essential factor affecting the effect of early life stress in adolescents. It was reported that adolescent female rats, not males that were exposed to social instability stress, displayed a transient increase in immobility in the forced swimming test during adolescence that diminished in adulthood (McCormick, Smith, & Mathews, 2008). The nature and severity of adverse stress, genders, and the strain of rodents are all critical elements that determine the outcomes of the effects of early life stress during adolescence (Freund, Thompson, DeNormandie, Vaccarro, & Andersen, 2013; Schroeder et al., 2018; Vetulani, 2013), and these factors may contribute to the variability of the previous results. Further studies need to clarify the roles of these factors in regulating the effect of early life stress in both adolescence and adulthood.

In the present study, we used C57BL/6 mouse models of MS and restraint stress (RS) to examine the effect of early life stress on anxiety‐related and depression‐like behaviors in adolescents and the sex difference. We also made a comparison of the results in adolescence and adulthood. MS and RS were applied alone or in combination at different time points during adolescence and adulthood. Open field and elevated plus maze tests were used to evaluate anxiety‐related behaviors, and forced swimming test for depression‐like behaviors.

## 2 MATERIALS AND METHODS

### 2.1 Animals

**Figure 1:**
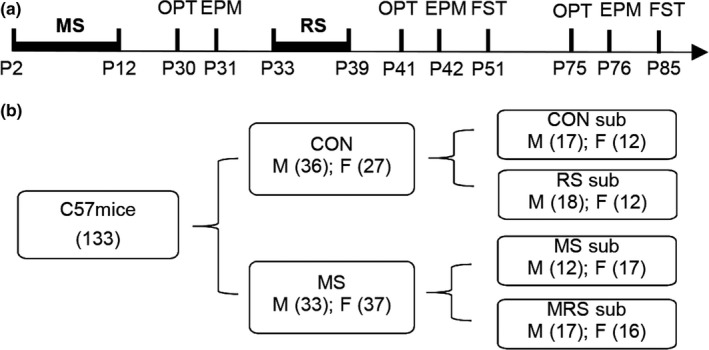
Schematic of the experiments. (A) The timeline of the behavioral tests. B57BL/6 mice were subjected to open field test (OFT) on P30, P41, and P75, and to elevated plus maze (EPM) on P31, P42, and P76. They were also subjected to the forced swimming test (FST) at two time points, P51 and P85. Maternal separation and restrain stress were performed from P2 to P12 and from P33 to P39, respectively. (B) A diagram for grouping mice. The values in parentheses denote the numbers of mice

Male and female C57BL/6 mice (6–8 weeks of age, 20–30 g) were purchased from Tengxing Biotechnology Company. Breeding was started after 1 week of their arrival in the facility. Each cage housed one male and two females, and the females were removed and housed individually after 10 days. Pregnant females were checked daily, and the date of giving birth was designated as postnatal day 0 (P0). All animals were housed in an acclimatized room at a temperature of around 22°C and on a 12‐hr light/dark cycle (08:00–20:00/20:00–08:00) with food and water ad libitum. The experimental schedule and the grouping of mice were shown in Figure 1. All the procedures in this study were approved by the Ethics Committee of Southwest University and followed the requirements of the National Institutes of Health Guidelines for Animal research.

### 2.2 Maternal separation

Litters were at first randomly assigned to the maternal separation (MS) group or the control (CON) group. For the MS group, pups were separated from their dams and kept individually in containers in a new room for 4 hr per day (10:00–14:00) from P2 to P12 (Figure 1a,b). The temperature in the containers was kept at 30–32°C using a heating pad. Dams remained in home cages, and pups were returned to home cages after separation. The CON group was reared under animal facility rearing conditions. Beddings were changed, and cages were cleaned every 5 days. Pups were weighed every 2 days for both the MS group and the control group. They were weaned at PD 21 and kept in cages with 4 mice per cage with free access to food and water.

### 2.3 Restraint stress

The mice that were subjected to restraint stress (RS) were placed into 50 ml plastic centrifuge tubes with holes at the tip for ventilation, and their movement was mostly limited. The mice remained in the tubes 4 hr per day (10:00–14:00) for 7 consecutive days from P33 to P39. RS was done in the homeroom. Weight was measured for each mouse every other day.

### 2.4 Behavioral tests

All behavioral tests were conducted between 09:00 and 16:00. Mice were transferred to a behavioral testing room and habituated to the testing room for 10 min before starting tests. The open field test, elevated plus maze and forced swimming test were performed with a video tracking system (EthoVision version 8.5, Noldus).

#### 2.4.1 Open field test

Open field test (OFT) was performed for all mice three times at ages of P30, P41, and P75. A box used for OFT was made of an opaque plastic board with an area of 40 × 40 cm and a wall in the height of 30 cm. The field was evenly illuminated with a dim red light. The area was divided into the central zone of 20 × 20 cm and the surrounding zone. Each mouse was placed in a corner and allowed to explore for 5 min. The ambulation of mice was recorded by a video camera (WV‐CP500/CH) connected to a computer.

#### 2.4.2 Elevated plus maze

Elevated plus maze (EPM) was performed three times for all mice at ages of P31, P42, and P76, respectively. The maze was made of opaque white plastic consisting of four perpendicularly intersected arms, and each arm was 5 cm wide and 32.5 cm long. Two arms had a wall with a height of 15 cm (closed arms), and the two others had no wall (open arms). The open and closed arms were connected by a central area (5 × 5 cm). The maze was elevated 40 cm from the floor. In the test, mice were placed in the central area, facing one of the open arms, and were recorded for 5 min.

#### 2.4.3 Forced swimming test

Forced swimming test (FST) was applied to all mice for two times at ages of PD51 and PD85. Each mouse was individually placed into a 13 cm × 25 cm (diameter × height) plastic cylinder filled with water with a depth of 10 cm at a temperature of 23–25°C for 6 min. After FST, the mice were removed from the water, gently dried, and returned to their home cages. Floating was defined as the smallest movement necessary to keep mice's head above the water. The percentage of immobility during the last 4 min was recorded.

### 2.5 Statistical analysis

Statistical analyses were performed using GraphPad Prism 6 (GraphPad Software). Data were presented as mean ± *SEM*. For the data with the *n* number equal to or more than 30, an unpaired *t* test was used to make comparisons between two groups and ANOVA for more than two groups, followed by Tukey's multiple comparison tests. For the data with the *n* number <30, the Kolmogorov–Smirnov (KS) test was firstly used to determine the normality of the data, and Brown–Forsythe test (for one‐way ANOVA) or *F* test (*t* test) was used to determine the homogeneity of variance of the data. For data that have normality and equal variance, unpaired *t* test or ANOVA were used. Otherwise, KS test or Kruskal–Wallis test (K‐W test) followed by Dunn's multiple comparisons were used. Two‐way repeated measures (rm) ANOVA was used for bodyweight analysis, followed by Bonferroni's or Tukey's multiple comparisons test. Statistical significance was set as *p* < .05.

## 3 RESULTS

### 3.1 The effect of early life stress on weight

**Figure 2:**
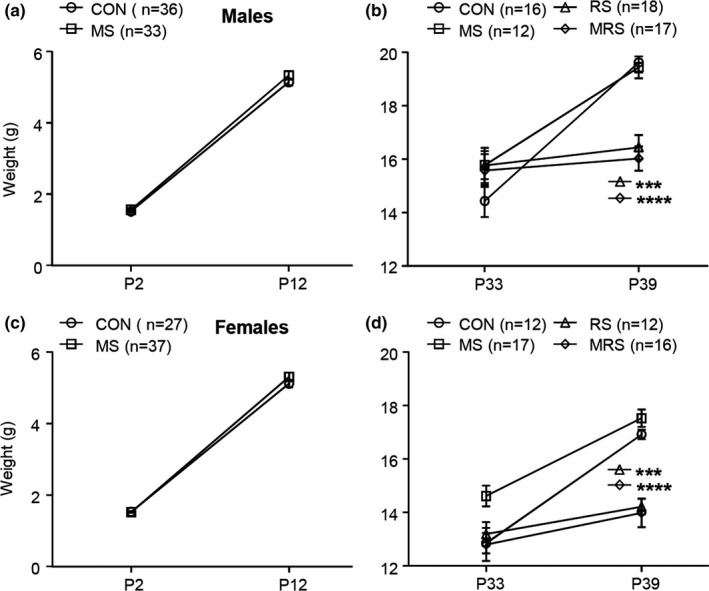
The effect of early life stress on weight. (A and C) Maternal separation (MS) has no effect on the weight gain both for males and females. Restraint stress (RS) and combined RS and MS (MRS) significantly reduce the weight gain both for males and females (B and D). Results are expressed as the mean ± SEM. Stars indicate the significant difference (*** *p*<0.001 and **** *p*<0.000)

As shown in Figure 2a,c, the weight of the mice on P12 was not significantly different between the CON group and the MS group both for males and females (*F*(1, 66) = 2.01, *p* = .16; post hoc Bonferroni's test: *p* > .99 for both males and females on P2, and *p* = .17 for males and *p* = .22 for females on P12, two‐way rmANOVA), and there was also no interaction between age and MS (*F*(1, 66) = 1.07, *p* = .30 for males; *F*(1, 63) = 1.62, *p* = .21 for females, two‐way rmANOVA). Two‐way rmANOVA analysis of the change of the weight from P33 to P39 for all four subgroups showed the significant difference both in males and females (*F*(3, 59) = 2.58, *p* = .06 for males; *F*(3, 53) = 10.20, *p* = .000 for females). Post hoc Tukey's test showed that the weight of the RS and the MS plus RS (MRS) subgroups on P39 in both males and females was significantly lower than the CON subgroup (*p* = .000 for males and females, Figure 2b,d). There was a significant interaction between age and stressors (*F*(3, 59) = 35.20, *p* = .000 for males, *F*(3, 53) = 16.60, *p* = .000 for females; two‐way rmANOVA).

### 3.2 The effect of early life stress on anxiety in adolescent mice

**Figure 3:**
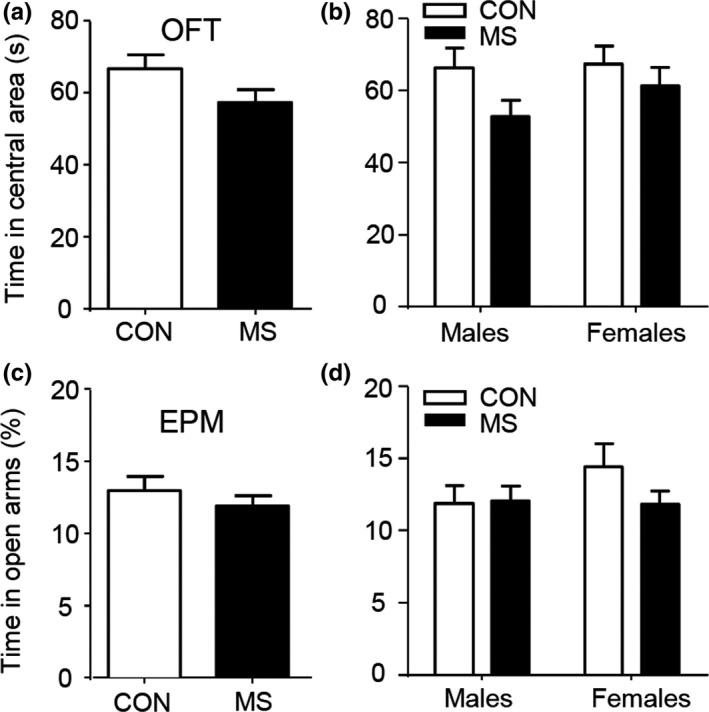
MS does not affect anxiety‐related behaviors in adolescent mice. (A) The time in the central area in the open field test (OFT) shows no difference between the MS group and the control groups of both sexes. (B) Same as (A) but separated by sex. No effect was found for all stressors in either males or females. (C) The percent time in open arms in the elevated plus maze (EPM) shows no difference between the MS group and the control group. (D) Same as (C) but separated by sex. No difference was seen in either sex

As illustrated in Figure 3, litters were randomly divided into two general groups of both sexes, the CON group (*n* = 63) with routine care, and MS group ( *n* = 70). No significant difference was found between the two groups in the time spent in the central area in OFT tested on P30 and the percent time spent in open arms in EPM tested on P31 (OFT, *t*_131_ = 1.82, *p* = .07; EPM, *t*_131_ = 0.85; *p* = .37; unpaired *t* test, Figure 3a,c). There was also no significant difference between two groups in both tests when the data were analyzed separately by sex (OPT: *p* = .24 for males, and *p* = .85 for females; EPM: *p* = .99 for males, and *p* = .43 for females, post hoc Tukey's test, two‐way ANOVA; Figure 3b,d). There was no interaction between sex and MS (OPT: *F*(1, 129) = 0.52, *p* = .47; EPM: *F*(1, 129) = 1.37, *p* = .24).

**Figure 4:**
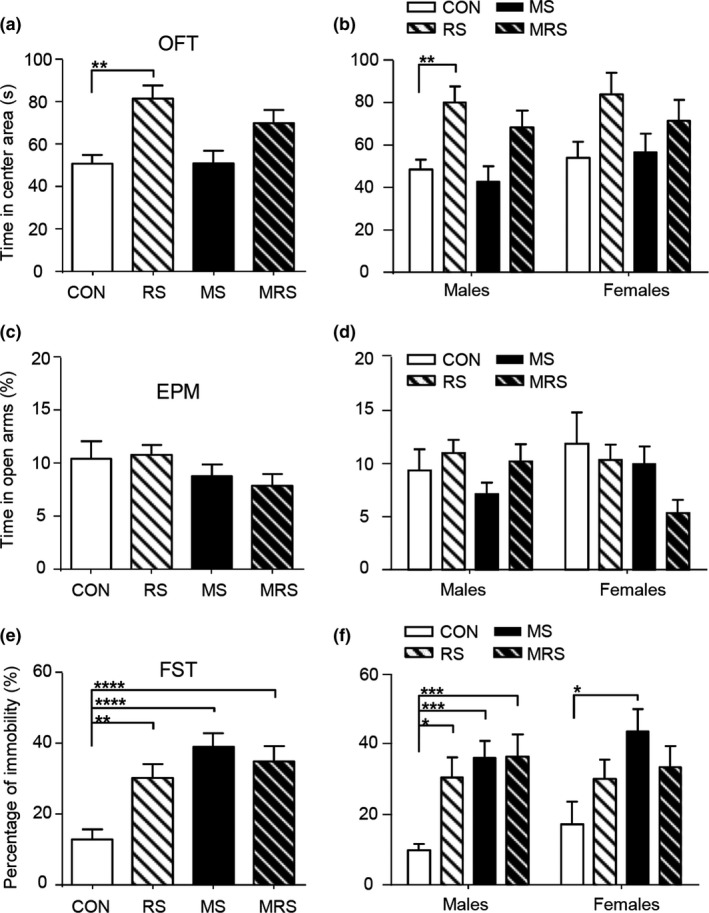
The effect of multiple stressors on the anxiety and depressive behaviors in adolescent mice. (a) The time spent in the central area in OFT tested in P41 adolescent mice of both sexes. RS significantly increases the time spent in the central area. (b) Same as (a) but separated by sex. RS significantly increases the time in males. (c) The percent time spent in open arms in EPM tested in adolescent mice of both sexes aged P42. No difference is seen among the four subgroups. (d) Same as (c) but separated by sex. No difference is seen among all subgroups in either males or females. (e) The percentage of immobility in FSTperformed in adolescent mice of both sexes on P51. All stressors significantly increase the percentage of immobility. (f) Same as (e) but separated by sex. In males, all stressors increase the percentage of immobility; in females, only MS increases it. Stars indicate the significance of the difference (*p < .05, **p < .01, ***p < .001, ****p < .0001)

We then examined the effect of RS alone or a combination of RS and MS, MRS, on anxiety‐related behaviors. As shown in Figure 1b, the CON group was further randomly divided into CON subgroup (*n* = 29) and RS subgroup (*n* = 30), and the MS group into MS subgroup (*n* = 29) and MRS subgroup (*n* = 33). All four subgroups were subjected to OFT and EPM again on P41 and P42, respectively. In OPT, compared to the CON subgroup of both sexes, only RS subgroup spent more time in the central area, and no difference was found in other subgroups (*F*(3, 117) = 6.89, *p* = .0003, post hoc Tukey's test: RS vs. CON, *p* = .002; one‐way ANOVA, Figure 4a). When the analysis was done for males and females separately, we found that the male RS subgroup spent more time in the central area in OPT (males, *F*(3, 60) = 5.81, *p* = .0015, post hoc Tukey's test: CON vs. RS: *p* = .0049; females, *F*(3,53) = 2.03, *p* = .12, one‐way ANOVA, Figure 4b). There was no interaction between sex and stress (*F*(3, 113) = 0.18, *p* = .91, two‐way ANOVA). In EPM, no significant difference was found among four subgroups of both sexes (*F*(3, 117) = 1.28, *p* = .29, one‐way ANOVA; Figure 4c). No interaction was found between sex and stress (*F*(3, 113) = 2.21, *p* = .09, two‐way ANOVA) and no main effect of stress was also found (*F*(3, 113) = 1.48, *p* = .23, Figure 4d).

### 3.3 The effect of stress on depression‐like behaviors in adolescent mice

All subgroups underwent FST on P51. Compared to the CON subgroup with both sexes, all other subgroups had a significantly higher percentage of immobility (*F*(3, 117) = 8.82, *p* < .000, one‐way ANOVA; *p* < .01 for all pairs, post hoc Tukey's test; Figure 4e). When data were analyzed separately by sex, no interaction was found between sex and stress (*F*(3, 113) = 0.48, *p* = .69, two‐way ANOVA). In males, all RS, MS, and MRS subgroups had a higher percentage of immobility (*F*(3, 60) = 6.879, *p* = .0005 post hoc Turkey's test, *p* = .000; one‐way ANOVA). In females, only MS subgroup had higher immobility (*F*(3, 53) = 2.73, *p* = .05; CON vs. MS subgroups, *p* = .039, post hoc Turkey's test; Figure 4f).

### 3.4 Effect of early life stress on anxiety‐related and depression‐like behaviors in adult mice

**Figure 5:**
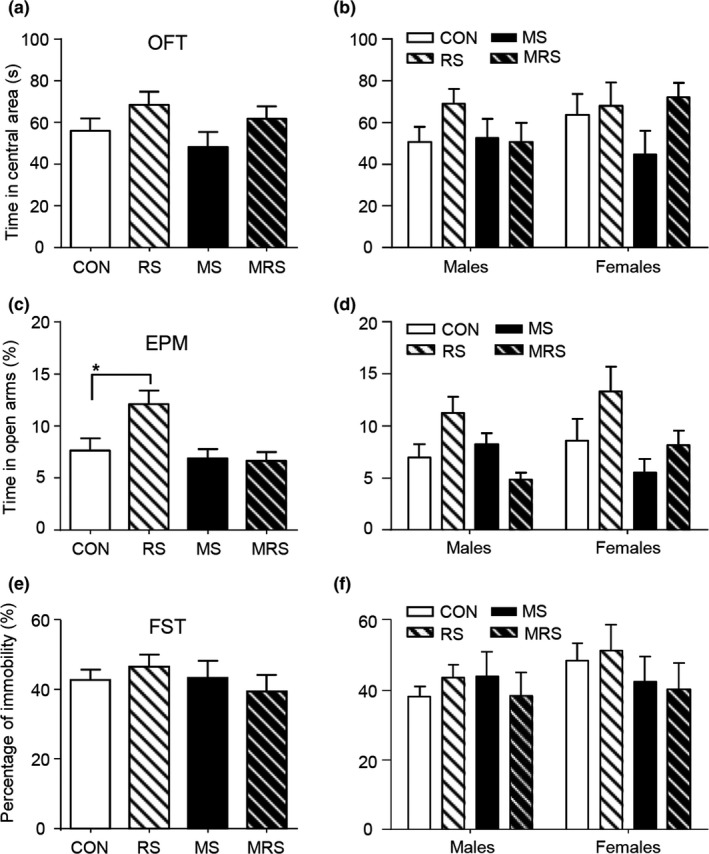
The effect of multiple stressors on the anxiety and depressive behaviors in adult mice. (a) The time spent in the central area in OFT performed in mice of both sexes aged P75. No difference was seen among all subgroups. (b) Same as (a) but separated by sex. No significant effect is seen relative to the CON subgroup. (c) The percent time in open arms in EPM in mice of both sexes aged P76. RS increases the percent time. (d) Same as (c) but separated by sex. No effect was seen in either males or females when compared to the CON subgroup. (e) The percentage of immobility in FST in mice of both sexes aged P85. All stressors have no significant effect. (f) Same as (e) but separated by sex. All stressors had no significant effect in either sex compared with the CON subgroup. The star indicates the significance of the difference (*p* < .05)

In order to know whether early life stress affects adolescents and adults differently, all subgroups were subjected to OPT and EPM on P75 and P76, respectively, and FST on P85. In OPT, compared to the CON subgroup in both sexes (*n* = 24), no significant difference in the time spent in central area was found in the MS (*n* = 22), RS (*n* = 18), and MRS (*n* = 24) subgroups (*F*(3, 84) = 1.74, *p* = .16, one‐way ANOVA; Figure 5a). When data were analyzed separately by sex, no interaction between sex and stress was found (*F*(3, 79) = 1.05, *p* = .37, two‐way ANOVA). And no effect of stress was also found (*F*(3, 79) = 1.56, *p* = .20; Figure 5b). In EPM test, the percent time in open arms in RS subgroup of both sexes was significantly higher compared to the CON subgroup (*F*(3, 89) = 5.77, *p* = .001, post hoc Tukey's test: RS vs. CON: *p* = .02; one‐way ANOVA; Figure 5c). There was no interaction between sex and stress (*F*(3, 85) = 1.32, *p* = .27, two‐way ANOVA). However, when males and females were analyzed separately, no difference was found in any subgroup in either sex compared to the CON subgroup (*p* > .1 for all pairs for males, Dunn's post hoc test, K‐W test and *p* > .3 for females, Tukey's post hoc test, one‐way ANOVA, Figure 5d).

As illustrated in Figure 5e, no significant difference in the percentage of immobility in FST was found among four subgroups of both sexes (*F*(3, 70) = 0.48, *p* = .70, one‐way ANOVA).

There was also no sex‐specific effect in all subgroups (*F*(1, 66) = 1.25, *p* = .27 for sex; *F*(3, 66) = 0.60, *p* = .62 for stress, two‐way ANOVA, Figure 5f), and no interaction between sex and stress (*F*(3, 66) = 0.49, *p* = .69, two‐way ANOVA).

## 4 DISCUSSION

In the present study, we used C57BL/6 mice and employed multiple types of stress, maternal separation (MS), restraint stress (RS), and their combination (MRS) to examine the effect of early life stress on anxiety‐related and depression‐like behaviors in adolescence and the sex difference. Our results showed that MS did not affect the anxiety level in adolescence for both males and females. RS decreased anxiety‐related behaviors in adolescent males, but not females. MS, RS, and MRS all significantly increased depression‐like behavior in male adolescents, but only MRS increased depression‐like behavior in female adolescents. The effect of these early life stress on depression‐like behavior in adolescence did not persist into adulthood.

Adolescence is a period of increased behavioral and psychiatric vulnerabilities and dramatic structural and functional neurodevelopment (Buwalda, Geerdink, Vidal, & Koolhaas, 2011; Sturman & Moghaddam, 2011). A small number of studies have directly examined the effect of early life stress on anxiety‐related and depression‐like behaviors in adolescent mice, and the results varied (Bian et al., 2015; Gracia‐Rubio et al., 2016; Iñiguez et al., 2014; Macrì & Laviola, 2004; Marco, Adriani, Llorente, Laviola, & Viveros, 2009; Shin et al., 2019, 2016). In our present study, MS was applied to the mice from P2 to P12 with 4 hr separation from the dams per day. This MS protocol is commonly used in mice and rats (Millstein & Holmes, 2007; Nylander & Roman, 2013; Vetulani, 2013). Our result that MS enhanced depressive behaviors but did not affect anxiety‐related behaviors in adolescent C57BL/6 mice is in consistence with some reports but different from others. In C57BL/6 adolescent mice, it was reported that MS given from P2 to P21 enhanced depression‐like behaviors in adolescent mice (Bian et al., 2015), but with the similar protocol and the same strain of mice, other studies reported that MS only increased anxiety‐related and aggressive behaviors in adolescents, not affecting depression‐like behavior (Shin et al., 2019, 2016). It was reported that additional stress after MS was needed to facilitate the effect of MS. MS (from P2 to P16, 3 hr per day or P7 to P15, 6 hr per day) with early weaning at P17, which was assumed to produce more stress than MS alone, enhanced anxiety‐related and depression‐like behaviors in C57BL/6 and CD‐1 adolescent mice (Gracia‐Rubio et al., 2016; Portero‐Tresserra et al., 2018; Tchenio, Lecca, Valentinova, & Mameli, 2017). In our study, we found RS alone decreased anxiety‐related behaviors in adolescence, but RS in addition to MS had no effect on the anxiety, which indicated that MS and RS might have different effects on anxiety‐related behaviors in adolescence. These variable results were likely attributed to the difference in the protocols of MS, especially the duration. Other factors, such as handling procedures and baseline stress levels, may also play roles (Bian et al., 2015; Shin et al., 2019, 2016).

The effect of MS in adolescence was also reported in rat models. Like in mouse models, the results in rat models also varied. Increased and decreased anxiety‐related behaviors, increased depression‐like behaviors, or no effect on anxiety‐related and depression‐like behaviors were all reported (Banqueri et al., 2017; Bian et al., 2015; Leussis et al., 2012; Lukkes et al., 2017; Wang et al., 2015; Yoo et al., 2012). The inconsistency may result from variable MS protocols used in different studies. MS given during the first 2 weeks after birth is a commonly used protocol in rat models, but other protocols were also adopted. For example, a single and prolonged episode (24 hr) of maternal deprivation at P9 or P12 facilitated depressive‐like symptoms during both adolescence and adulthood (Marco et al., 2009). It was found in rats that MS from P1 to P10, 4 hr per day led to decreased associative/emotional learning only in adolescence, but MS (P1 to P21) increased anxious behavior and impaired recognition in both adolescence and adulthood (Banqueri et al., 2017). It is evident that the duration and timing of MS were both crucial factors in manifesting the MS effect. Further studies are needed to understand the mechanisms underlying the varied effects of different MS protocols.

The effect of MS in adulthood have been extensively studied in mice and rats, and the results were diverse, even contradicting (Tractenberg et al., 2016). In one study, eight different strains of mice were used to examine the effect of MS in adults, and it was found the typic protocol of MS that pups were separated from their dams for 3 hr per day from P0‐P13 did not significantly affect the anxiety and depressive level in all strains (Millstein & Holmes, 2007). Another work studied two protocols of MS, one separating pups from their dams from P2 to P14, 3 hr per day, and another using the same protocol of MS but with increasing hours of separation plus an early weaning at P17, showed that both protocols had no significant effect on anxiety‐related and depression‐like behaviors in adults (Tan, Ho, Song, Low, & Je, 2017). These results are in contrast with many other studies using a variety of MS protocols in mice showing the effect of MS on anxiety‐related and depression‐like behaviors in adulthood (Lesse, Rether, Gröger, Braun, & Bock, 2017; Romeo et al., 2003; Tchenio et al., 2017). In those above studies, anxiety and depression were tested in adulthood, not in adolescence; thus, it is not known if MS has any transient effect in adolescence. Our results indicated that MS had a transient effect in adolescence, especially in male adolescents. It is possible that the intensity of our MS protocol may not be strong enough to affect the anxiety‐related and depression‐like behaviors in adults. It is also possible that adolescent depression‐like behaviors are mechanistically different from ones in adulthood.

Some works indicated that additional stress in adolescence or adulthood is needed to manifest the effect of MS (Hill et al., 2014; Marais, van Rensburg, van Zyl, Stein, & Daniels, 2008; Vargas, Junco, Gomez, & Lajud, 2016). In our study, RS in adolescence increased depression‐like behavior only in males but not females. Our results are consistent with a report in male C57BL/6 mice that social defeat stress‐induced depression‐like behaviors in adolescence (Iñiguez et al., 2014). RS in mice subjected to MS did not induce a higher level of depressive behavior, indicating that RS and MS may share a similar pathway for inducing depression‐like behaviors. Similar results were found in the rats that RS did not enhance the MS effect on depression (van Zyl et al., 2016).

Female adolescents have a more than doubled risk of developing major depressive disorder relative to males (Avenevoli et al., 2015). It has been indicated that adolescence is a critical developmental period highly susceptible to adverse stress, especially for females. In rodent models, the effect of MS has been studied mostly in male adults (Huot, Plotsky, Lenox, & McNamara, 2001). Our present work also revealed the sex‐specific effect of early life stress. RS decreased the anxiety level in adolescent males, but not females. MS, RS, and MRS all increased depression‐like behavior in adolescent males, but only MRS increased depression‐like behavior in female adolescents. It indicated that females were more resistant to stress in terms of depression‐like behavior, which is in agreement with a previous report in rat models that female rats were resistant to MS‐induced depression‐like behavior (Dimatelis, Vermeulen, Bugarith, Stein, & Russell, 2016). In the female, the estrous cycle may affect the effect of MS. It was reported that MS led to increased anxiety in males, but in females only when they were in the diestrous phase of their estrous cycle (Romeo et al., 2003). Since our test was done in females without considering the estrous cycle, our results might not reflect the possible effect of the estrous cycle. The sex‐specific effect of MS may also depend on the strains of mice, for it was shown that MS induced a more evident deficit in recognition memory in females (de Azeredo et al., 2017) in BALB/c mice. It was also reported that MS affected males and females in different manners, impairing controllability in an escapable shock in males, but motivation in the no‐shock condition in females and the effect in females was no longer seen in adulthood (Leussis et al., 2012).

## 5 CONCLUSION

The present results indicate that early life stress affects anxiety‐related and depression‐like behaviors in adolescent mice in a manner specific to sexes and stressors. These effects are transient and occurred during adolescence. Previous studies have resulted in significant variability in the effect of early life stress in rat and mouse models. Multiple factors might contribute to the variability, especially the differences in the protocols, gender, strain, and timing. Experimental conditions and handlings may be additional factors. Further work under strictly controlled conditions is needed to validate the animal models of early life stress further.

## CONFLICT OF INTEREST

None declared.

## Data Availability

The data that support the findings of this study are available from the corresponding author upon reasonable request.
